# Depressive symptoms and risk of incident activities of daily living disability among older adults with symptomatic arthritis

**DOI:** 10.3389/fmed.2026.1796800

**Published:** 2026-06-01

**Authors:** Jingxuan Cui, Jian Kang, Shuaiyu Li, Linru Zeng, Chen Li

**Affiliations:** 1 The First School of Clinical Medicine, Zhejiang Chinese Medical University, Hangzhou, China; 2 Jiangnan Hospital Affiliated to Zhejiang Chinese Medical University, Hangzhou, China; 3 The Third Affiliated Hospital of Zhejiang Chinese Medical University, Hangzhou, China

**Keywords:** arthritis, daily living disability, depressive symptoms, risk of incident activities, symptomatic arthritis

## Abstract

**Background:**

Depressive symptoms are highly prevalent among older adults with symptomatic arthritis and may contribute to accelerated functional decline. However, large-scale longitudinal evidence regarding the association between depression and the onset of activities of daily living (ADL) disability remains limited, particularly when competing mortality risks and potential mediating pathways are taken into account. This study aimed to examine the relationship between depressive symptoms and incident ADL disability among older adults with symptomatic arthritis using two nationally representative cohorts.

**Methods:**

This longitudinal cohort study analyzed data from the US Health and Retirement Study (HRS) and the English Longitudinal Study of Ageing (ELSA). Multivariable Cox proportional hazards models, Fine-Gray competing risk regression models, and discrete-time logistic regression models with follow-up waves as the time scale were used to evaluate the association between depressive symptoms and ADL disability risk. Restricted cubic spline (RCS) analyses were conducted to assess potential non-linear associations. Mediation analyses were performed to quantify the mediating role of physical activity.

**Results:**

During follow-up, 484 participants in ELSA and 1,525 participants in HRS developed incident ADL disability. In both cohorts, higher Center for Epidemiologic Studies Depression Scale (CES-D) scores were significantly associated with an increased risk of ADL disability. In fully adjusted Cox models, each 1-point increase in CES-D score was associated with a 7% higher risk of ADL disability in ELSA (adjusted HR = 1.07, 95% CI: 1.02–1.11) and a 10% higher risk in HRS (adjusted HR = 1.10, 95% CI: 1.07–1.12). Compared with participants without depression, those with depression had a substantially elevated risk of ADL disability (ELSA: adjusted HR = 1.26, 95% CI: 1.04–1.54; HRS: adjusted HR = 1.49, 95% CI: 1.33–1.67). These associations remained robust in competing risk models and discrete-time logistic regression analyses. RCS analyses revealed a dose–response relationship between depressive symptoms and ADL disability risk, with evidence of non-linearity in the HRS cohort. Subgroup analyses demonstrated consistent associations across demographic and clinical subgroups. Mediation analyses indicated that physical activity partially mediated the association between depressive symptoms and ADL disability, accounting for approximately 9.1% of the total effect in ELSA and 5.0% of that in HRS.

**Conclusion:**

In both the United States and the United Kingdom, depressive symptoms were independently associated with an increased risk of ADL disability among older adults with symptomatic arthritis. This relationship was robust across multiple analytical frameworks and exhibited a clear dose–response pattern. Physical activity partially mediated this association, highlighting the potential value of integrated mental health and lifestyle interventions to mitigate functional decline in this vulnerable population.

## Introduction

Disability in activities of daily living (ADL) constitutes a major public health challenge in ageing societies worldwide. ADL disability is highly prevalent among older adults; however, reported estimates vary considerably depending on age, population characteristics, and measurement definitions. Meta-analytic evidence suggests a prevalence of approximately 15–25% among community-dwelling older adults, whereas substantially higher rates—approaching 40–50%—have been reported in older subgroups (e.g., aged ≥75 years) and in studies adopting broader definitions of functional impairment (1–4). These variations highlight the importance of contextualizing prevalence estimates when comparing across populations. Regardless of the specific estimate, ADL disability profoundly compromises quality of life and independence, increases healthcare utilization and costs, and is associated with an elevated mortality risk.

Symptomatic arthritis, encompassing osteoarthritis and rheumatoid arthritis, is highly prevalent in older populations, affecting up to one-third of individuals aged 65 years or older. Prevalence increases with advancing age and is consistently higher among women (5, 6). Arthritis contributes to functional decline and ADL disability primarily through chronic pain, joint stiffness, and reduced mobility, which interfere with essential daily activities, such as bathing, dressing, and walking (7). Persistent pain and stiffness exacerbate muscle weakness and joint instability, fostering a self-reinforcing cycle of physical inactivity and progressive functional impairment (8). Importantly, however, functional outcomes among individuals with symptomatic arthritis are heterogeneous. Not all affected individuals develop ADL disability, and substantial variability exists, influenced by factors such as comorbid conditions, treatment adherence, physical activity levels, and psychosocial characteristics. This heterogeneity highlights the importance of identifying modifiable determinants beyond arthritic pathology (9, 10).

Depressive symptoms are also common in later life, with prevalence estimates varying widely depending on assessment tools and population characteristics. In community-dwelling older adults, prevalence is often reported in the range of 15–30%; however, among individuals with chronic conditions such as arthritis, substantially higher rates have been observed. Prior studies indicate that the prevalence of depressive symptoms in individuals with arthritis or functional limitations frequently exceeds 30–40% (11–15). In the present study, the prevalence of depression—defined using the eight-item Center for Epidemiologic Studies Depression Scale (CES-D; cutoff ≥3)—was approximately 29.8% in ELSA and 27.2% in HRS, which is broadly consistent with existing literature when accounting for differences in measurement instruments and study populations. These findings underscore the clinical and public health importance of examining depression as a potential contributor to functional decline.

Despite growing interest in this area, several critical gaps persist. Many prior studies have failed to account for death as a competing risk, potentially biasing estimates of ADL disability incidence by ignoring differential mortality among high-risk individuals (16). In addition, limited use of complementary modeling strategies across continuous and discrete time frameworks has introduced uncertainty regarding the robustness of temporal associations (17). Evidence on non-linear or dose–response relationships between depression severity and ADL disability risk is also scarce, constraining refined risk stratification and clinical translation (18). Furthermore, empirical investigations of mediating mechanisms, particularly the role of physical activity in linking depression to functional outcomes, remain limited, hindering the development of targeted and mechanism-informed interventions (19). Collectively, these limitations underscore the need for rigorous longitudinal research to clarify underlying pathways and inform effective prevention strategies.

To address these gaps, the present study utilized data from two large, nationally representative longitudinal cohorts—the Health and Retirement Study (HRS) and the English Longitudinal Study of Ageing (ELSA)—to examine the association between depressive symptoms and incident ADL disability among older adults with symptomatic arthritis. A comprehensive analytical framework was applied, including Cox proportional hazards models, competing risk analyses, discrete-time survival models, restricted cubic spline analyses to assess non-linearity, subgroup analyses, and mediation analyses. The use of these complementary methods was intended to strengthen causal inference, enhance robustness, and improve the interpretability of the findings.

## Materials and methods

### Study population

This study employed a parallel, harmonized, multi-cohort longitudinal design using data from the Health and Retirement Study (HRS) and the English Longitudinal Study of Ageing (ELSA). The two cohorts were analyzed independently using identical inclusion criteria, variable definitions, and statistical models, rather than being combined into a single pooled dataset.

The primary objective of this design was cross-cohort validation, allowing assessment of the robustness, consistency, and generalizability of the association between depressive symptoms and incident ADL disability across two nationally representative populations from different healthcare and sociocultural contexts.

Baseline waves were selected to maximize temporal alignment and data comparability (Wave 7 for HRS and Wave 2 for ELSA). Follow-up proceeded prospectively within each cohort until the occurrence of incident ADL disability, death, loss to follow-up, or the end of available observation. All analyses were conducted separately in HRS and ELSA, and results were subsequently compared qualitatively in terms of direction, magnitude, and statistical significance.

**Figure 1:**
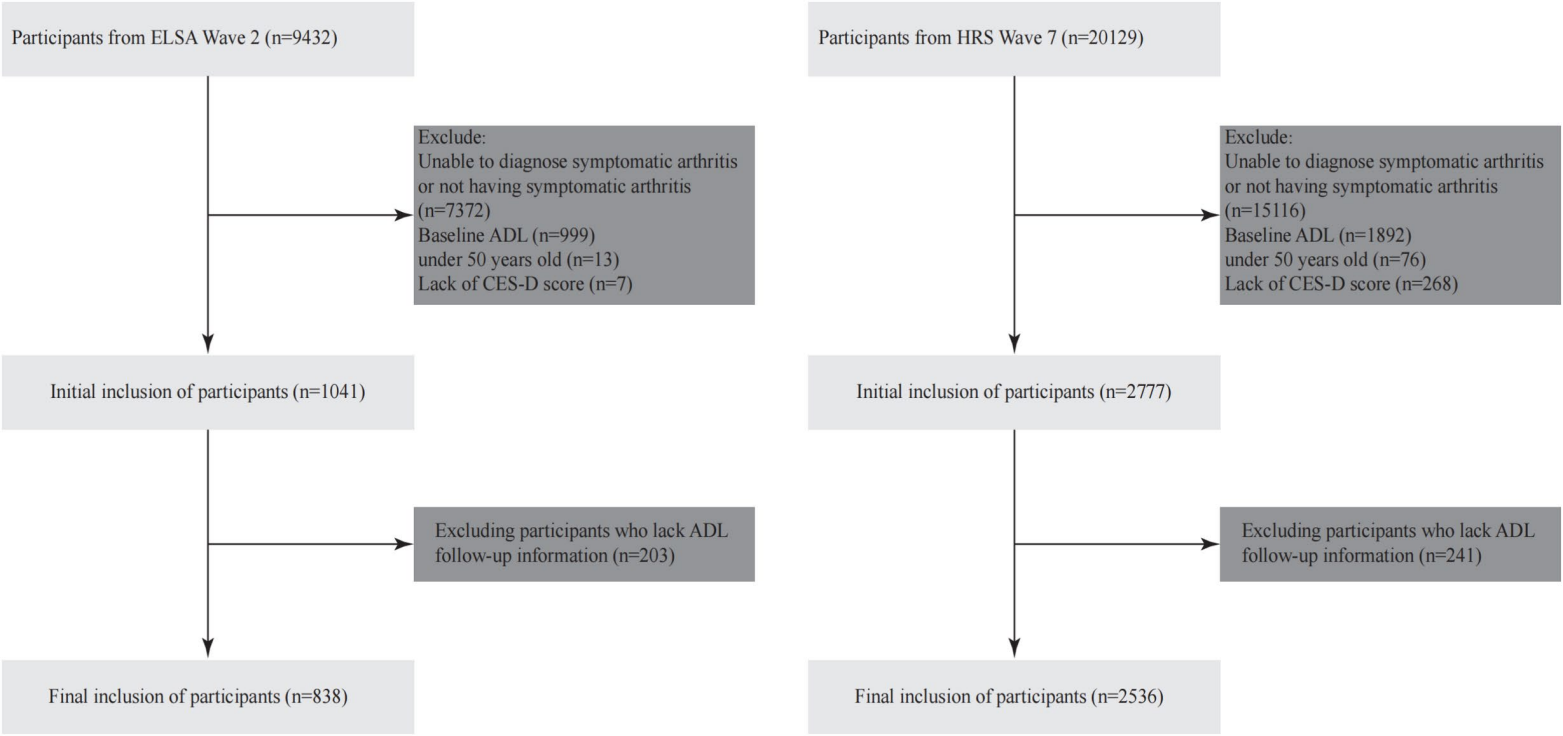
Flowchart of participant selection.

In the ELSA cohort, 9,432 participants were initially identified. After applying predefined exclusion criteria, 8,594 participants were excluded, including those without symptomatic arthritis at baseline (*n* = 7,372), those with prevalent ADL disability at baseline (*n* = 999), those younger than 50 years (*n* = 13), those with missing baseline Center for Epidemiologic Studies Depression Scale (CES-D) scores (*n* = 7), and those lacking follow-up ADL information (*n* = 203). The final analytical sample from ELSA comprised 838 participants (Figure 1).

In the HRS cohort, 20,129 participants were initially identified. Of these, 17,593 were excluded because of the absence of symptomatic arthritis at baseline (*n* = 15,116), baseline ADL disability (*n* = 1,892), age younger than 50 years (*n* = 76), missing baseline CES-D scores (*n* = 268), or missing follow-up ADL data (*n* = 241). The final analytical sample included 2,536 participants from the HRS cohort (Figure 1).

### Assessment of depressive symptoms

Depressive symptoms were assessed at baseline using the eight-item version of the Center for Epidemiologic Studies Depression Scale (CES-D). This abbreviated scale has been widely used in large population-based studies, including HRS and ELSA, and has demonstrated good validity for capturing depressive symptom burden in older adults. Total scores range from 0 to 8, with higher scores indicating greater symptom severity. Consistent with prior studies, a cutoff score of ≥3 was used to define elevated depressive symptoms, which reflects clinically relevant depressive symptomatology rather than a formal diagnosis of major depressive disorder (20).

### Assessment of symptomatic arthritis

Participants with arthritis were identified based on self-reported physician diagnosis in both the HRS and ELSA cohorts. To approximate symptomatic arthritis, we included individuals who reported arthritis in conjunction with functional limitations or mobility-related difficulties, reflecting the presence of clinically relevant symptoms affecting daily functioning. This operational definition is consistent with prior epidemiological studies using large population-based datasets in which detailed clinical assessments of disease activity are not available. However, it should be noted that this definition does not distinguish between different arthritis subtypes or does not capture objective measures of disease severity.

### Assessment of ADL disability

ADL reflects the essential physical tasks required for independent functioning. In both HRS and ELSA, ADL were assessed using the following six items: dressing, bathing, eating, getting in and out of bed, using the toilet, and walking across a room. Each activity was scored as 1 if the participant reported difficulty performing the task. Scores were summed across items, and participants with a total score ≥ 1 were defined as having ADL disability (21).

### Assessment of physical activity

Physical activity was assessed using self-reported measures in both the HRS and ELSA cohorts. In HRS, participants were asked about the frequency of engagement in moderate and vigorous physical activities, such as gardening, walking at a moderate pace, sports, or exercise. Similarly, in ELSA, respondents reported how often they participated in moderate or vigorous physical activities, including activities such as brisk walking, cycling, or exercise. Physical activity was defined as engagement in moderate or vigorous physical activity at least once per week, based on harmonized self-reported questionnaire items from HRS and ELSA.

To ensure comparability across cohorts, physical activity was harmonized and operationalized as a dichotomous variable. Participants were classified as physically active if they reported engaging in moderate or vigorous physical activity at least once per week, and as physically inactive otherwise. This definition is consistent with prior epidemiological studies utilizing HRS and ELSA data.

The classification incorporates both moderate and vigorous activity levels; however, detailed information on duration, metabolic equivalent (MET) intensity, or structured exercise participation was not consistently available across both cohorts. Therefore, further stratification by intensity or total volume was not feasible in the present study.

In the mediation analysis, physical activity was treated as a binary mediator (active versus inactive) to examine its potential role in the pathway linking depressive symptoms to incident ADL disability. While this approach allows for cross-cohort harmonization, it may not fully capture the complexity of physical activity behaviors, and this limitation has been acknowledged.

### Covariates

Covariates included sociodemographic and lifestyle factors as well as clinical conditions. These variables comprised age, sex, ethnicity, education, wealth, body mass index (BMI), smoking status, drinking status, hypertension, diabetes, physical activity, and cardiovascular disease. Hypertension was defined as a self-reported physician diagnosis of high blood pressure and current use of antihypertensive medication. Diabetes was defined as a self-reported physician diagnosis. Smoking status was categorized as non-smoker or current smoker, and drinking status as non-drinker or current drinker. BMI was calculated as weight (kg) divided by height squared (m^2^).

### Statistical analyses

All statistical analyses were performed separately for the HRS and ELSA cohorts following an identical analytical pipeline. No pooled analyses were conducted. This approach was chosen to avoid biases arising from differences in sampling frames, measurement distributions, and healthcare systems, while enabling independent replication of findings across cohorts. Consistency of associations across cohorts was evaluated by comparing effect directions, effect sizes, confidence intervals, dose–response patterns, and mediation proportions, rather than formal statistical pooling. Concordant findings across cohorts were interpreted as evidence of robustness and external validity.

Continuous variables were summarized as means and standard deviations, whereas categorical variables were presented as counts and weighted percentages. Between-group differences were evaluated using Student’s t tests or chi-square tests, as appropriate. Multivariable Cox proportional hazards regression models were used to estimate the association between depressive symptoms and the risk of incident ADL disability among participants with symptomatic arthritis. Hazard ratios (HRs) and 95% confidence intervals (CIs) were calculated with sequential adjustment for potential confounders. Model 1 was unadjusted. Model 2 was adjusted for age, sex, ethnicity, education, marital status, wealth, and BMI. Model 3 was further adjusted for hypertension, diabetes, smoking status, and drinking status. Model 4 additionally included physical activity and cardiovascular disease.

Given that death may preclude the occurrence of ADL disability, competing risk analyses were performed using the Fine–Gray subdistribution hazard model, with death treated as a competing event. Subdistribution hazard ratios (sHRs) and 95% CIs were reported to assess the robustness of the associations under a competing risk framework. To further examine the stability of the findings under an alternative time specification, discrete-time logistic regression models were fitted using follow-up waves as the time unit. Data were reshaped into a person–period format, with each participant contributing one observation per follow-up wave until the first occurrence of ADL disability or censoring.

Restricted cubic spline (RCS) analyses were conducted to explore potential non-linear associations between depressive symptom scores and ADL disability risk.

Subgroup analyses were conducted to examine whether the association between depressive symptoms (CES-D score) and incident ADL disability varied across key demographic characteristics. The analyses were stratified by age, sex, and ethnicity, which are established determinants of both depression and functional decline. In these analyses, depressive symptoms were treated as the exposure variable, and separate models were fitted within each subgroup. All subgroup analyses were based on fully adjusted models, controlling for sociodemographic factors, health behaviors, and comorbid conditions.

A mediation analysis was conducted to explore whether physical activity may partially mediate the association between depressive symptoms and incident ADL disability. The primary models were adjusted for potential confounders. To assess the robustness of the findings, sensitivity analyses were performed using unadjusted mediation models without the inclusion of covariates. This approach allows for evaluation of the extent to which covariate adjustment influences the estimated indirect effects. The mediation analysis relies on several assumptions, including no unmeasured confounding of the exposure–outcome, exposure–mediator, and mediator–outcome relationships, as well as correct model specification. Given the observational nature of the data, these assumptions cannot be fully verified.

The selection of physical activity as a mediator was guided by *a priori* conceptual considerations. Depressive symptoms may influence functional decline through behavioral pathways, particularly reduced engagement in physical activity, which has been consistently associated with both depression and disability in older adults. Therefore, physical activity was considered a plausible mediator linking depressive symptoms to incident ADL disability. In contrast, socioeconomic factors (e.g., wealth and education) and comorbid conditions (e.g., hypertension, diabetes, and cardiovascular disease) were treated as confounders and included as covariates in the models. These variables are generally regarded as upstream determinants that may influence both depressive symptoms and functional outcomes, rather than mediators in the causal pathway examined in this study.

To further examine potential dose–response relationships, CES-D scores were additionally categorized into ordinal groups (0, 1–2, 3–4, and ≥5), representing increasing levels of depressive symptom burden. All analyses were performed using IBM SPSS Statistics (version 24.0) and R software (version 4.3.0). A two-sided *p*-value of < 0.05 was considered statistically significant.

## Results

### Baseline characteristics according to ADL status among participants with symptomatic arthritis

Tables 1, 2 summarize baseline characteristics of participants with symptomatic arthritis stratified by ADL status in the ELSA and HRS cohorts, respectively. In the ELSA cohort, 838 participants without ADL disability at baseline were included. The mean age was 67.07 years (SD = 9.44), and 575 participants (68.62%) were women. Over a median follow-up of 10 years, 484 participants developed incident ADL disability, with a mean age of 68.15 years (SD = 9.29). Compared with those who remained free of ADL disability, participants who developed ADL disability were older (68.15 vs. 65.59 years, *p* < 0.001), had lower household wealth (206,831.99 vs. 275,660.71, *p* < 0.001), higher BMI (29.22 vs. 27.94, *p* < 0.001), and a higher prevalence of hypertension (52.48% vs. 47.52%, *p* = 0.020) and depression (32.85% vs. 25.71%, *p* = 0.026) (Table 1).

**Table 1:** Baseline characteristics of participants stratified by incident activities of daily living (ADL) disability in the ELSA cohort.

Characteristic	*N*	Overall *N* = 838	No ADL disability *N* = 354	ADL disability *N* = 484	*p*-value
Sex^b^, *n* (%)	838				0.285
Female		575 (68.62%)	250 (70.62%)	325 (67.15%)	
Male		263 (31.38%)	104 (29.38%)	159 (32.85%)	
Age (year)^a^, Mecan ± SD	838	67.07 ± 9.44	65.59 ± 9.44	68.15 ± 9.29	<0.001
Ethnicity^b^, *n* (%)	838				0.902
No white		16 (1.91%)	7 (1.98%)	9 (1.86%)	
White		822 (98.09%)	347 (98.02%)	475 (98.14%)	
Education^b^, *n* (%)	838				0.451
Before high school		477 (56.92%)	200 (56.50%)	277 (57.23%)	
High school		145 (17.30%)	58 (16.38%)	87 (17.98%)	
Junior college		139 (16.59%)	57 (16.10%)	82 (16.94%)	
College graduate or above		77 (9.19%)	39 (11.02%)	38 (7.85%)	
Wealth^a^, Mean ±	838	235,907.60 ± 320,213.44	275,660.71 ± 399,596.48	206,831.99 ± 242,862.81	<0.001
BMI^a^, Mean ±	838	28.68 ± 4.68	27.94 ± 4.25	29.22 ± 4.91	<0.001
Marital status^b^, *n* (%)	838				0.072
Married		560 (66.83%)	255 (72.03%)	305 (63.02%)	
Separation		6 (0.72%)	3 (0.85%)	3 (0.62%)	
Divorced		81 (9.67%)	29 (8.19%)	52 (10.74%)	
Widowed		166 (19.81%)	57 (16.10%)	109 (22.52%)	
Never married		25 (2.98%)	10 (2.82%)	15 (3.10%)	
Hypertension^b^, *n* (%)	838				0.020
No		427 (50.95%)	197 (55.65%)	230 (47.52%)	
Yes		411 (49.05%)	157 (44.35%)	254 (52.48%)	
Diabetes^b^, *n* (%)	838				0.034
No		774 (92.36%)	335 (94.63%)	439 (90.70%)	
Yes		64 (7.64%)	19 (5.37%)	45 (9.30%)	
Smoking status^b^, *n* (%)	838				0.288
No		307 (36.63%)	137 (38.70%)	170 (35.12%)	
Yes		531 (63.37%)	217 (61.30%)	314 (64.88%)	
Drinking status^b^, *n* (%)	838				0.228
No		185 (22.08%)	71 (20.06%)	114 (23.55%)	
Yes		653 (77.92%)	283 (79.94%)	370 (76.45%)	
Physical activity^b^, *n* (%)	838				0.012
No		222 (26.49%)	78 (22.03%)	144 (29.75%)	
Yes		616 (73.51%)	276 (77.97%)	340 (70.25%)	
Cardiovascular disease^b^, *n* (%)	838				0.875
No		625 (74.58%)	265 (74.86%)	360 (74.38%)	
Yes		213 (25.42%)	89 (25.14%)	124 (25.62%)	
CES-D^a^, Mean ± SD	838	2.01 ± 2.12	1.77 ± 1.97	2.19 ± 2.21	0.004
CES-D (non-depressed)^a^, Mean ± SD		1.09 ± 1.00	1.05 ± 1.00	1.12 ± 1.00	0.035
CES-D (depressed)^a^, Mean ± SD		5.67 ± 1.25	5.67 ± 1.23	5.67 ± 1.27	0.057
Depression^b^, *n* (%)	838				0.026
No		588 (70.17%)	263 (74.29%)	325 (67.15%)	
Yes		250 (29.83%)	91 (25.71%)	159 (32.85%)	

^a^Student’s *t*-test, ^b^Chi-square test, SD: standard deviation. Wealth was measured in country-specific currencies (USD in HRS and GBP in ELSA).

**Table 2:** Baseline characteristics of participants stratified by incident activities of daily living (ADL) disability in the HRS cohort.

Characteristic	*N*	Overall *N* = 2,536	No ADL disability *N* = 1,011	ADL disability *N* = 1,525	*p*-value
Sex^b^, *n* (%)	2,536				0.013
Female		1,678 (66.17%)	640 (63.30%)	1,038 (68.07%)	
Male		858 (33.83%)	371 (36.70%)	487 (31.93%)	
Age (year)^a^, Mean ± SD	2,536	67.02 ± 9.43	64.66 ± 8.65	68.58 ± 9.61	<0.001
Ethnicity^b^, *n* (%)	2,536				0.001
Non-Hispanic white		2,022 (79.73%)	845 (83.58%)	1,177 (77.18%)	
Non-Hispanic Blacks		291 (11.47%)	98 (9.69%)	193 (12.66%)	
Hispanic		172 (6.78%)	52 (5.14%)	120 (7.87%)	
Other ethnicities		51 (2.01%)	16 (1.58%)	35 (2.30%)	
Education^b^, *n* (%)	2,536				<0.001
High school or less		616 (24.29%)	193 (19.09%)	423 (27.74%)	
Some college		1,499 (59.11%)	641 (63.40%)	858 (56.26%)	
College graduate or above		421 (16.60%)	177 (17.51%)	244 (16.00%)	
Wealth^a^, Mean ±	2,536	396,544.89 ± 1,097,342.38	447,248.28 ± 1,401,920.94	362,931.03 ± 835,242.43	<0.001
BMI^a^, Mean ±	2,536	28.32 ± 5.69	27.94 ± 5.42	28.58 ± 5.85	0.022
Marital status^b^, *n* (%)	2,536				<0.001
Married		1,648 (64.98%)	720 (71.22%)	928 (60.85%)	
Separation		53 (2.09%)	19 (1.88%)	34 (2.23%)	
Divorced		296 (11.67%)	112 (11.08%)	184 (12.07%)	
Widowed		468 (18.45%)	129 (12.76%)	339 (22.23%)	
Never married		71 (2.80%)	31 (3.07%)	40 (2.62%)	
Hypertension^b^, *n* (%)	2,536				<0.001
No		1,066 (42.03%)	466 (46.09%)	600 (39.34%)	
Yes		1,470 (57.97%)	545 (53.91%)	925 (60.66%)	
Diabetes^b^, *n* (%)	2,536				0.002
No		2,082 (82.10%)	860 (85.06%)	1,222 (80.13%)	
Yes		454 (17.90%)	151 (14.94%)	303 (19.87%)	
Smoking status^b^, *n* (%)	2,536				0.074
No		1,025 (40.42%)	387 (38.28%)	638 (41.84%)	
Yes		1,511 (59.58%)	624 (61.72%)	887 (58.16%)	
Drinking status^b^, *n* (%)	2,536				<0.001
No		1,357 (53.51%)	491 (48.57%)	866 (56.79%)	
Yes		1,179 (46.49%)	520 (51.43%)	659 (43.21%)	
Physical activity^b^, *n* (%)	2,536				<0.001
No		752 (29.65%)	234 (23.15%)	518 (33.97%)	
Yes		1,784 (70.35%)	777 (76.85%)	1,007 (66.03%)	
Cardiovascular disease^b^, *n* (%)	2,536				<0.001
No		1798 (70.90%)	762 (75.37%)	1,036 (67.93%)	
Yes		738 (29.10%)	249 (24.63%)	489 (32.07%)	
CES-D^a^, Mean ± SD	2,536	1.81 ± 2.06	1.45 ± 1.86	2.05 ± 2.15	<0.001
CES-D (non-depressed)^a^, Mean ± SD		0.99 ± 1.03	0.86 ± 0.98	1.08 ± 1.05	<0.001
CES-D (depressed)^a^, Mean ± SD		5.52 ± 1.36	5.50 ± 1.33	5.53 ± 1.37	0.034
Depression^b^, *n* (%)	2,536				<0.001
No		1,846 (72.79%)	812 (80.32%)	1,034 (67.80%)	
Yes		690 (27.21%)	199 (19.68%)	491 (32.20%)	

^a^Student’s *t*-test, ^b^Chi-square test, SD: standard deviation. Wealth was measured in country-specific currencies (USD in HRS and GBP in ELSA).

In the HRS cohort, 2,536 participants with symptomatic arthritis and no baseline ADL disability were included. The mean age was 67.02 years (SD = 9.43), with 1,678 women (66.17%). During a median follow-up of 6 years, 1,525 participants developed incident ADL disability, with a mean age of 68.58 years (SD = 9.61). Participants who developed ADL disability were older (68.58 vs. 64.66 years, *p* < 0.001), had lower household wealth (362,931.03 vs. 447,248.28, *p* < 0.001), higher BMI (28.58 vs. 27.94, *p* = 0.022), and were more likely to have hypertension (60.66% vs. 39.34%, *p* < 0.001) and depression (32.20% vs. 19.68%, *p* < 0.001) than those without incident ADL disability (Table 2).

Although the mean CES-D scores in the overall sample were below the cutoff value, this reflects the distribution of depressive symptoms in community-based populations. Additional analyses stratified by depression status demonstrated clear differences in CES-D levels between depressed and non-depressed participants within ADL groups (Tables 1, 2).

### Association between depression and risk of ADL disability

Table 3 presents the associations between depressive symptoms and incident ADL disability based on Cox proportional hazards models. In unadjusted analyses, higher CES-D scores were significantly associated with an increased risk of ADL disability in both cohorts (ELSA: HR = 1.08, 95% CI: 1.04–1.12, *p* < 0.001; HRS: HR = 1.12, 95% CI: 1.09–1.14, *p* < 0.001). These associations remained statistically significant after full adjustment for sociodemographic factors, lifestyle behaviors, and comorbidities (Model 4) (ELSA: adjusted HR = 1.07, 95% CI: 1.02–1.11, *p* = 0.003; HRS: adjusted HR = 1.10, 95% CI: 1.07–1.12, *p* < 0.001). Each 1-point increase in CES-D score was associated with a 7% higher risk of ADL disability in ELSA and a 10% higher risk in HRS.

**Table 3:** Associations between depressive symptoms and the risk of incident ADL disability among individuals with symptomatic arthritis based on Cox proportional hazards models.

Exposure variable	Model 1		Model 2		Model 3		Model 4	
	HR (95% CI)	*p*-value	HR (95% CI)	*p*-value	HR (95% CI)	*p*-value	HR (95% CI)	*p*-value
ELSA								
CES-D	1.08 (1.04–1.12)	<0.001	1.07 (1.02–1.11)	0.002	1.07 (1.02–1.11)	0.003**	1.07 (1.02–1.11)	0.003
Depression								
No	1.00 (Reference)	–	1.00 (Reference)	–	1.00 (Reference)	–	1.00 (Reference)	–
Yes	1.37 (1.13–1.66)	0.001	1.27 (1.04–1.54)	0.017	1.26 (1.03–1.53)	0.022	1.26 (1.04–1.54)	0.021
*p* for trend	1.08 (1.03–1.13)	0.001	1.06 (1.01–1.11)	0.017	1.06 (1.01–1.11)	0.022	1.06 (1.01–1.11)	0.021
HRS								
CES-D	1.12 (1.09–1.14)	<0.001	1.11 (1.08–1.14)	<0.001	1.11 (1.08–1.13)	<0.001	1.10 (1.07–1.12)	<0.001
Depression								
No	1.00 (Reference)	–	1.00 (Reference)	–	1.00 (Reference)	–	1.00 (Reference)	–
Yes	1.71 (1.54–1.91)	<0.001	1.56 (1.40–1.74)	<0.001	1.54 (1.38–1.73)	<0.001	1.49 (1.33–1.67)	<0.001
*p* for trend	1.20 (1.15–1.24)	<0.001	1.16 (1.12–1.20)	<0.001	1.16 (1.11–1.20)	<0.001	1.14 (1.10–1.19)	<0.001

Model 1: Crude. Model 2: Adjust: age, sex, ethnicity, education, marital status, wealth, BMI. Model 3: Adjust: age, sex, ethnicity, education, marital status, wealth, BMI, hypertension, diabetes, drinking status, and smoking status. Model 4: Adjust: age, sex, ethnicity, education, marital status, wealth, BMI, hypertension, diabetes, drinking status, smoking status, physical activity, and cardiovascular disease.

When depression was defined using a CES-D cutoff of ≥ 3, participants with depression had a significantly higher risk of ADL disability than those without depression after multivariable adjustment (ELSA: adjusted HR = 1.26, 95% CI: 1.04–1.54, *p* = 0.021; HRS: adjusted HR = 1.49, 95% CI: 1.33–1.67, *p* < 0.001). Trend analyses further demonstrated that increasing CES-D quartiles were associated with progressively higher risks of ADL disability in both cohorts (ELSA: adjusted HR = 1.06, 95% CI: 1.01–1.11, *p* = 0.021; HRS: adjusted HR = 1.14, 95% CI: 1.10–1.19, *p* < 0.001) (Table 3).

Analyses using ordinal CES-D categories showed a graded association, with higher levels of depressive symptoms associated with progressively increased risk of incident ADL disability (*p* for trend < 0.001), consistent with the primary analyses (Supplementary Table 1).

**Figure 2:**
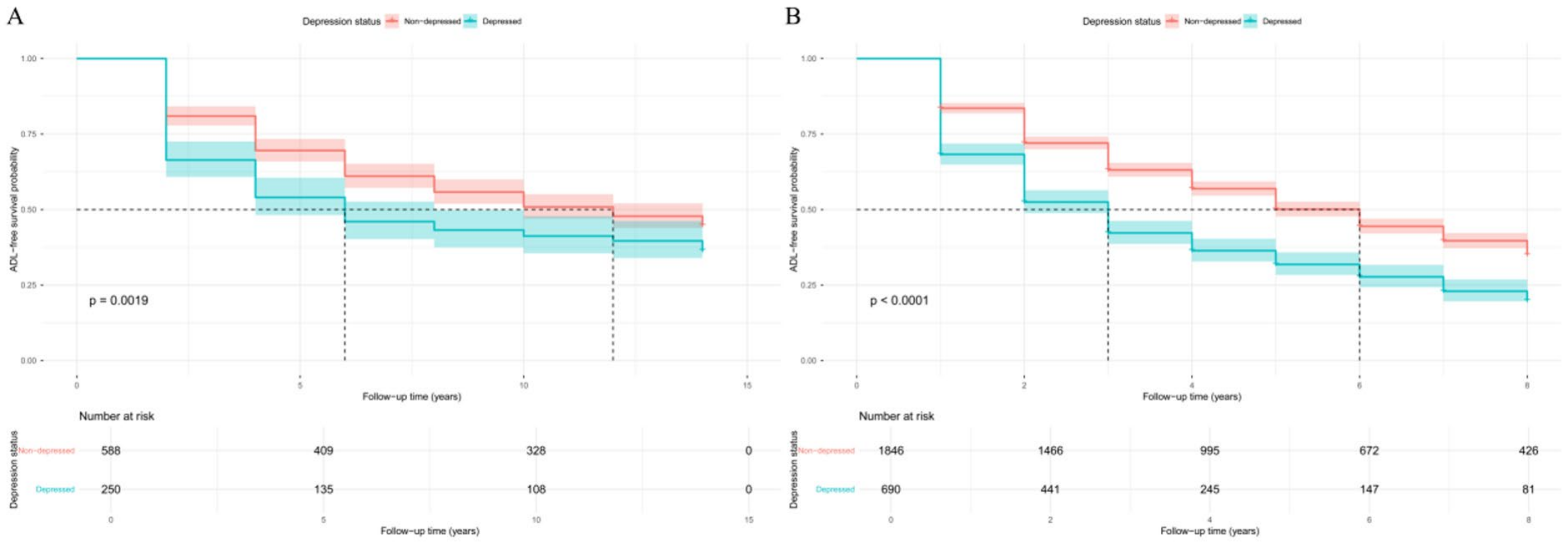
Kaplan–Meier curves for ADL disability–free survival according to depression status: **(A)** ELSA cohort; **(B)** HRS cohort.

Kaplan–Meier curves showed significantly lower ADL disability-free survival among participants with depression compared with those without depression in both cohorts (ELSA: log-rank *p* = 0.0019; HRS: log-rank *p* < 0.001). The divergence between survival curves increased over time (Figure 2).

### Competing risk analysis accounting for death

The results from Fine–Gray competing risk regression analyses are presented in Table 4. After accounting for death as a competing event, depressive symptoms remained independently associated with a higher subdistribution hazard of ADL disability. In fully adjusted models, CES-D scores were positively associated with ADL disability risk (ELSA: adjusted sHR = 1.06, 95% CI: 1.02–1.10, *p* = 0.003; HRS: adjusted sHR = 1.09, 95% CI: 1.06–1.11, *p* < 0.001). Participants with depression also exhibited a higher subdistribution hazard of ADL disability compared with those without depression (ELSA: adjusted sHR = 1.22, 95% CI: 1.02–1.47, *p* = 0.031; HRS: adjusted sHR = 1.43, 95% CI: 1.29–1.58, *p* < 0.001). Significant trends were observed across CES-D categories in both cohorts. The magnitude and direction of these associations were comparable to those observed in the Cox models, indicating a minimal influence of competing mortality risk (Table 4).

**Table 4:** Competing risk analysis of the association between depressive symptoms and incident ADL disability among individuals with symptomatic arthritis, with death treated as a competing event.

Exposure variable	Model 1		Model 2		Model 3		Model 4	
	sHR (95% CI)	*p*-value	sHR (95% CI)	*p*-value	sHR (95% CI)	*p*-value	sHR (95% CI)	*p*-value
ELSA								
CES-D	1.07 (1.03 ~ 1.11)	<0.001	1.06 (1.02 ~ 1.10)	0.003	1.06 (1.02 ~ 1.10)	0.003	1.06 (1.02 ~ 1.10)	0.003
Depression								
No	1.00 (Reference)	-	1.00 (Reference)	-	1.00 (Reference)	-	1.00 (Reference)	-
Yes	1.32 (1.10 ~ 1.57)	0.002	1.23 (1.03 ~ 1.48)	0.025	1.22 (1.02 ~ 1.46)	0.033	1.22 (1.02 ~ 1.47)	0.031
*p* for trend	1.07 (1.02 ~ 1.12)	0.002	1.05 (1.01 ~ 1.10)	0.025	1.05 (1.00 ~ 1.10)	0.033	1.05 (1.00 ~ 1.10)	0.031
HRS								
CES-D	1.11 (1.08 ~ 1.13)	<0.001	1.10 (1.07 ~ 1.12)	<0.001	1.09 (1.07 ~ 1.12)	<0.001	1.09 (1.06 ~ 1.11)	<0.001
Depression								
No	1.00 (Reference)	–	1.00 (Reference)	–	1.00 (Reference)	–	1.00 (Reference)	–
Yes	1.62 (1.47 ~ 1.79)	<0.001	1.49 (1.34 ~ 1.65)	<0.001	1.47 (1.33 ~ 1.63)	<0.001	1.43 (1.29 ~ 1.58)	<0.001
*p* for trend	1.18 (1.14 ~ 1.21)	<0.001	1.14 (1.10 ~ 1.18)	<0.001	1.14 (1.10 ~ 1.18)	<0.001	1.13 (1.09 ~ 1.16)	<0.001

Model 1: Crude. Model 2: Adjust: age, sex, ethnicity, education, marital status, wealth, and BMI. Model 3: Adjust: age, sex, ethnicity, education, marital status, wealth, BMI, hypertension, diabetes, drinking status, and smoking status. Model 4: Adjust: age, sex, ethnicity, education, marital status, wealth, BMI, hypertension, diabetes, drinking status, smoking status, physical activity, and cardiovascular disease.

### Discrete-time logistic regression analysis

Discrete-time logistic regression models using follow-up waves as the time unit yielded results consistent with those of the continuous-time analyses (Table 5). In fully adjusted models, higher CES-D scores were associated with increased odds of incident ADL disability within each follow-up interval (ELSA: adjusted OR = 1.11, 95% CI: 1.05–1.16, *p* < 0.001; HRS: adjusted OR = 1.11, 95% CI: 1.08–1.14, *p* < 0.001). Participants with depression had substantially higher odds of developing ADL disability than those without depression (ELSA: adjusted OR = 1.65, 95% CI: 1.30–2.09, *p* < 0.001; HRS: adjusted OR = 1.58, 95% CI: 1.39–1.81, *p* < 0.001). These findings further support the robustness of the observed associations across alternative time modeling strategies.

**Table 5:** Discrete-time logistic regression analysis of depressive symptoms and incident ADL disability using follow-up waves as the time unit.

Exposure variable	Model 1		Model 2		Model 3		Model 4	
	OR (95% CI)	*p*-value	OR (95% CI)	*p*-value	OR (95% CI)	*p*-value	OR (95% CI)	*p*-value
ELSA								
CES-D	1.11 (1.06 ~ 1.16)	<0.001	1.11 (1.05 ~ 1.16)	<0.001	1.10 (1.05 ~ 1.16)	<0.001	1.11 (1.05 ~ 1.16)	<0.001
Depression								
No	1.00 (Reference)	–	1.00 (Reference)	–	1.00 (Reference)	–	1.00 (Reference)	–
Yes	1.75 (1.40 ~ 2.18)	<0.001	1.66 (1.32 ~ 2.09)	<0.001	1.63 (1.29 ~ 2.06)	<0.001	1.65 (1.30 ~ 2.09)	<0.001
*p* for trend	1.15 (1.09 ~ 1.21)	<0.001	1.14 (1.07 ~ 1.20)	<0.001	1.13 (1.07 ~ 1.20)	<0.001	1.13 (1.07 ~ 1.20)	<0.001
HRS								
CES-D	1.13 (1.10 ~ 1.16)	<0.001	1.12 (1.09 ~ 1.15)	<0.001	1.12 (1.09 ~ 1.15)	<0.001	1.11 (1.08 ~ 1.14)	<0.001
Depression								
No	1.00 (Reference)	–	1.00 (Reference)	–	1.00 (Reference)	–	1.00 (Reference)	–
Yes	1.80 (1.59 ~ 2.03)	<0.001	1.66 (1.46 ~ 1.89)	<0.001	1.65 (1.45 ~ 1.88)	<0.001	1.58 (1.39 ~ 1.81)	<0.001
*p* for trend	1.15 (1.11 ~ 1.19)	<0.001	1.10 (1.06 ~ 1.15)	<0.001	1.10 (1.06 ~ 1.14)	<0.001	1.08 (1.04 ~ 1.12)	<0.001

Model 1: Crude. Model 2: Adjust: age, sex, ethnicity, education, marital status, wealth, and BMI. Model 3: Adjust: age, sex, ethnicity, education, marital status, wealth, BMI, hypertension, diabetes, drinking status, and smoking status.

### Subgroup analyses

**Figure 3:**
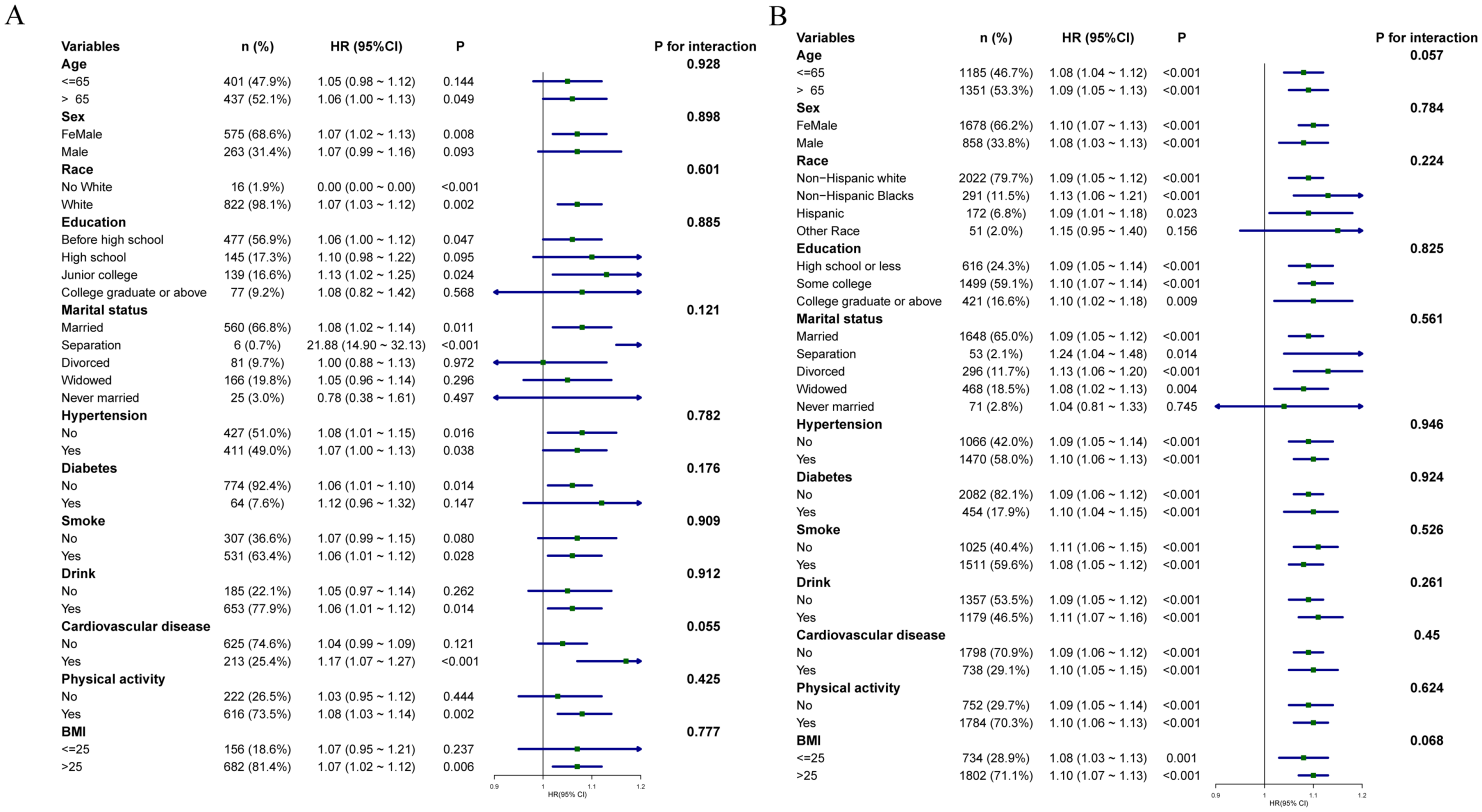
Subgroup analyses of the association between depressive symptoms (CES-D score) and incident ADL disability. Hazard ratios (HRs) and 95% confidence intervals (CIs) were estimated using fully adjusted Cox proportional hazards models within strata defined by age, sex, and ethnicity. Models were adjusted for sociodemographic characteristics, lifestyle factors, and major comorbid conditions. Depressive symptoms were treated as the exposure variable in all subgroup analyses.

Subgroup analyses demonstrated that the association between depression and ADL disability was largely consistent across strata defined by sex, age, hypertension, and other chronic conditions. No statistically significant interactions were detected between depression and subgroup variables (all *p* for interaction > 0.05), suggesting limited effect modification (Figure 3). Subgroup analyses demonstrated that the association between depressive symptoms and ADL disability was generally consistent across age, sex, and ethnicity groups, with no substantial evidence of heterogeneity (Supplementary Figure 1).

### Linear and non-linear associations between depression and ADL disability

**Figure 4:**
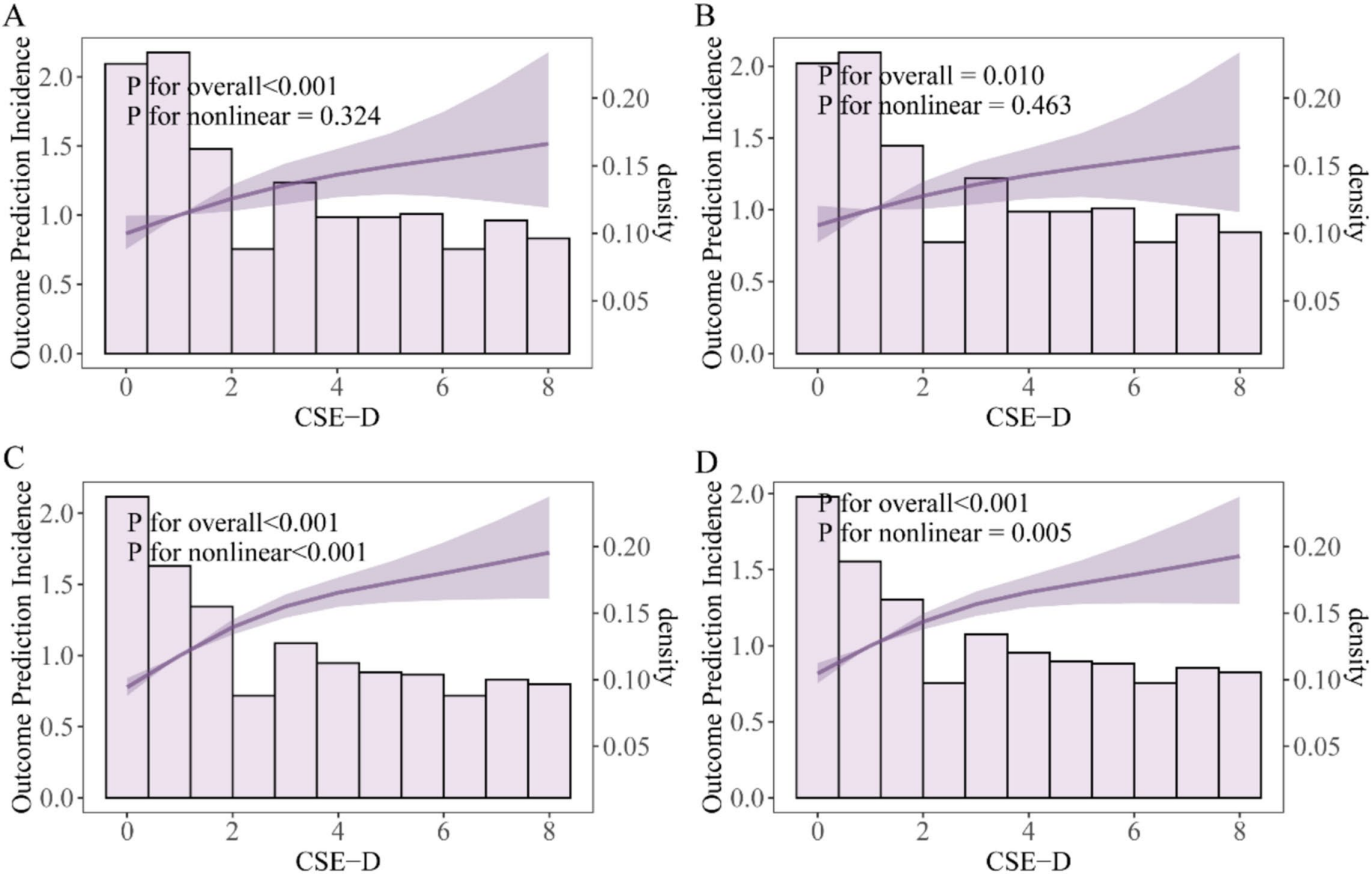
Restricted cubic spline analyses illustrating linear and non-linear associations between depression and the risk of incident ADL disability among individuals with symptomatic arthritis: **(A)** Unadjusted model, ELSA cohort; **(B)** Fully adjusted model, ELSA cohort; **(C)** Unadjusted model, HRS cohort; **(D)** Fully adjusted model, HRS cohort.

Restricted cubic spline analyses were conducted to characterize potential non-linear relationships between depressive symptoms and ADL disability risk. In the ELSA cohort, CES-D scores showed a linear association with ADL disability risk in both unadjusted (*p* for non-linearity = 0.324; *p* for overall < 0.001) and fully adjusted models (*p* for non-linearity = 0.463; *p* for overall = 0.010) (Figures 4A,B). In contrast, a significant non-linear association was observed in the HRS cohort in unadjusted analyses (*p* for non-linearity < 0.001; *p* for overall < 0.001), which persisted after adjustment for confounders (*p* for non-linearity = 0.005; *p* for overall < 0.001) (Figures 4C,D). Threshold analyses identified an inflection point at a CES-D score of 6.610. Below this value, CES-D scores were positively associated with ADL disability risk (adjusted HR = 1.13, 95% CI: 1.09–1.16, *p* < 0.001). Above the threshold, the association remained positive but was no longer statistically significant (adjusted HR = 1.38, 95% CI: 0.86–2.23, *p* = 0.180) (Table 6). These analyses were based on the continuous distribution of CES-D scores, allowing for assessment of dose–response relationships across the full range of depressive symptom burden rather than relying solely on predefined categorical thresholds.

**Table 6:** Threshold effect analysis of the non-linear association between depression and the risk of incident ADL disability among individuals with symptomatic arthritis in the HRS cohort.

Parameter	HR (95% CI), *p*-value
Model 1 Fitting model by standard linear regression	1.10 (1.07 ~ 1.12), <0.001
Model 2 Fitting model by two-piecewise linear regression	
Inflection point	6.610
<6.610	1.13 (1.09–1.16), <0.001
>6.610	1.38 (0.86–2.23), 0.180
*p* for likelihood ratio test	0.003

Model 1: Standard linear regression with CES-D as a continuous predictor. Model 2: Two-piecewise linear regression with CES-D split at the inflection point. Significance: *p* < 0.05 indicates statistical significance.

### Mediation analysis

**Figure 5:**
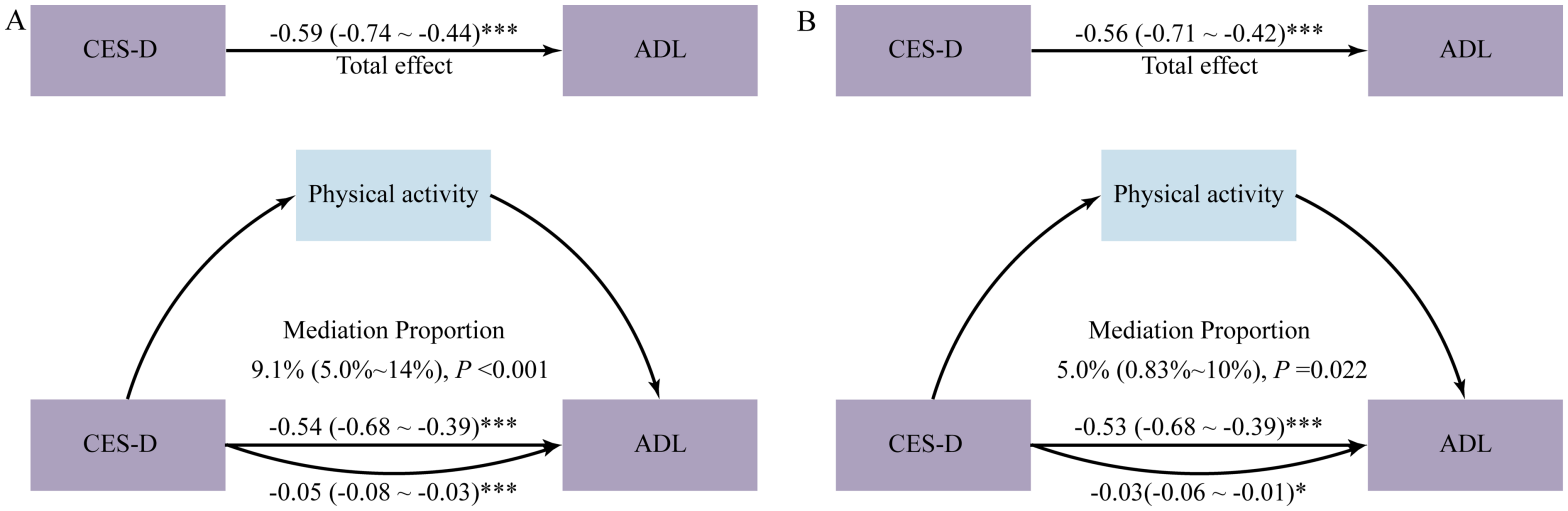
Mediation effect of physical activity on the association between depressive symptoms and incident ADL disability: **(A)** ELSA cohort; **(B)** HRS cohort.

Mediation analyses indicated that physical activity partially mediated the association between depressive symptoms and ADL disability (Figure 5). In the ELSA cohort, physical activity accounted for approximately 9.1% of the total effect, whereas in the HRS cohort it accounted for approximately 5.0%. In both cohorts, the direct and total effects of depressive symptoms on ADL disability remained statistically significant, suggesting that additional pathways beyond physical activity contribute to the observed associations. Sensitivity analyses using unadjusted mediation models yielded results consistent with those from the fully adjusted models, with physical activity demonstrating a modest but significant mediating effect in the association between depressive symptoms and incident ADL disability.

## Discussion

In this large longitudinal analysis of older adults with symptomatic arthritis, depressive symptoms were independently associated with a significantly increased risk of incident limitations in ADL. This association was consistently observed across multiple analytical frameworks, including Cox proportional hazards models, competing risk regression models accounting for death, and discrete-time logistic regression analyses. Notably, the relationship persisted after a comprehensive adjustment for sociodemographic characteristics, health-related behaviors, and major chronic conditions. Participants with higher depressive symptom scores, as well as those meeting criteria for clinically relevant depression, remained at substantially elevated risk of developing ADL impairment during follow-up. Furthermore, dose–response analyses revealed a graded increase in ADL risk with rising depressive symptom burden, supporting a hierarchical association rather than a simple threshold effect. Mediation analyses additionally suggested that physical activity may partially account for the observed relationship between depression and functional decline.

Differences in follow-up duration between the two cohorts may have influenced the observed associations. Compared with ELSA, which had a longer follow-up period of approximately 10 years, the HRS cohort had a shorter follow-up duration of approximately 6 years. Shorter follow-up periods may be more sensitive to capturing short-term effects of depressive symptoms on functional decline, whereas longer follow-up allows for the accumulation of risk over time and may better reflect long-term trajectories. This difference in temporal scale may partly explain the non-linear association observed in the HRS cohort, as early effects may be more pronounced, while longer follow-up in ELSA may have smoothed or attenuated such patterns. These findings highlight the importance of considering follow-up duration when interpreting cross-cohort differences.

Our findings are consistent with previous studies demonstrating that depression is an important contributor to functional impairment among individuals with arthritis. By applying a robust longitudinal design in a clearly defined high-risk population, the present study further extends the existing evidence base (22). Prior research has shown that depressive symptoms are associated with greater functional limitation in patients with rheumatoid arthritis, with pain and fatigue acting as key mediators, which accords with the associations observed here (14). Other studies have highlighted the bidirectional relationship between chronic conditions, such as arthritis and depression, whereby functional symptoms, including limitations in activities of daily living, can exacerbate depressive episodes. This observation closely aligns with our findings that identify depression as an independent risk factor for functional decline (23). By providing longitudinal evidence focused specifically on symptomatic arthritis, our study addresses limitations inherent in the predominantly cross-sectional designs used in this field and demonstrates consistency of results across countries. Collectively, these strengths enhance the generalizability of the findings to broader aging populations (24, 25).

The association between depressive symptoms and impairment in ADL among individuals with symptomatic arthritis likely reflects the interplay of behavioral, physiological, and psychological mechanisms. From a behavioral perspective, depression is commonly associated with reduced motivation, leading to lower levels of physical activity, poorer adherence to arthritis treatment regimens, and diminished engagement in social networks. Together, these factors accelerate functional deterioration (26, 27). From a physiological perspective, depressive states may exacerbate inflammatory processes characteristic of arthritis, disrupt neuroendocrine balance through elevated cortisol levels, and increase pain sensitivity via altered central nervous system processing. These changes may further aggravate joint stiffness and mobility limitations (28, 29). From a psychological perspective, depression amplifies the perceived burden of functional impairment, creating a self-reinforcing cycle in which subjective distress further restricts daily activities and sustains disability over time (15).

Mediation analysis indicated that physical activity partially mediated the association between depressive symptoms and ADL impairment, providing quantitative support for a behavioral pathway linking depression to functional decline (30). The selection of physical activity as a mediator was guided by *a priori* conceptual considerations, as reduced engagement in physical activity is a well-established consequence of depressive symptoms and a key determinant of functional capacity in individuals with arthritis. In contrast, socioeconomic factors and comorbid conditions were treated as confounders rather than mediators, as they are more appropriately conceptualized as upstream determinants influencing both depressive symptoms and disability outcomes.

Notably, the observed mediation effect was modest and remained consistent across both adjusted and unadjusted models, suggesting that the findings are relatively robust to model specification. However, physical activity represents only one of several potential pathways, and the incomplete mediation observed indicates that additional mechanisms—such as inflammation, pain amplification, or cognitive processes—are likely to contribute. Given the observational nature of the data and the potential for residual confounding and bidirectional relationships, these results should be interpreted as exploratory and hypothesis-generating rather than evidence of a definitive causal pathway (31, 32).

Several methodological strengths enhance the reliability of the present study relative to prior research. The use of two large, nationally representative longitudinal cohorts—the Health and Retirement Study and the English Longitudinal Study of Ageing—enabled extended follow-up and cross-cultural comparison, thereby minimizing biases inherent in smaller or geographically restricted samples (33). The integration of Cox proportional hazards models, Fine–Gray competing risk models, and discrete-time logistic regression allowed for comprehensive evaluation of time-dependent outcomes and potential biases, including competing mortality risk (34). The application of restricted cubic spline analyses further clarified non-linear and dose–response relationships, providing a more nuanced understanding than simple linear models (35). In addition, extensive subgroup and sensitivity analyses confirmed the stability of the findings across diverse population strata, strengthening confidence in their robustness and validity (36, 37).

Despite these strengths, several limitations should be acknowledged. The reliance on self-reported assessments of depressive symptoms and ADL may introduce measurement bias, including overreporting or underreporting influenced by mood or recall inaccuracy (38). Although extensive covariate adjustment was performed, residual confounding due to unmeasured factors, such as genetic susceptibility or environmental exposures, cannot be fully excluded (39). While informative, the mediation analysis depends on assumptions regarding the absence of unmeasured confounding, which may not be fully satisfied in an observational setting and therefore limits causal inference (40). Furthermore, because the study population consisted primarily of community-dwelling older adults, generalization to institutionalized individuals or younger populations should be approached with caution. These limitations do not undermine the principal conclusions but instead highlight important directions for future research, including the use of objective measures and the inclusion of more diverse populations. Additionally, physical activity was assessed using self-reported frequency-based measures and dichotomized for harmonization across cohorts, which may not fully capture differences in intensity, duration, or structured exercise patterns. Future studies using device-based or more granular measures are warranted to further elucidate the mediating role of physical activity.

## Conclusion

Across two nationally representative longitudinal cohorts, depressive symptoms were independently associated with an increased risk of incident ADL impairment among older adults with symptomatic arthritis. This association was robust across multiple analytical approaches, including Cox regression, competing risk models accounting for death, and discrete-time logistic regression, and it demonstrated a clear dose–response pattern. Mediation analyses further indicated that physical activity partially explains the link between depressive symptoms and subsequent functional decline, underscoring the role of behavioral pathways in this relationship. Collectively, these findings highlight the importance of early identification and management of depressive symptoms in older adults with symptomatic arthritis and suggest that integrated interventions targeting both mental health and physical activity may help reduce future functional deterioration.

## Data Availability

The datasets presented in this study can be found in online repositories. The names of the repository/repositories and accession number(s) can be found below: The original data for this study are available on their respective websites: The Health and Retirement Study-HRS (https://hrs.isr.umich.edu), the English Longitudinal Study of Ageing-ELSA (https://www.elsa-project.ac.uk).
