# Supplementary material for: Depressive symptoms and risk of incident activities of daily living disability among older adults with symptomatic arthritis
Source: Front Med (Lausanne). 2026 Jun 1;13:1796800. doi: 10.3389/fmed.2026.1796800 (PMC13330604; doi:10.3389/fmed.2026.1796800)

Supplementary Table 1. Associations between ordinal categories of depressive symptoms (CES-D: 0, 1–2, 3–4, ≥5) and the risk of incident ADL disability among individuals with symptomatic arthritis: results from Cox proportional hazards models in ELSA and HRS.

| Exposure Variable | Model 1 | | Model 2 | | Model 3 | | Model 4 | |
| --- | --- | --- | --- | --- | --- | --- | --- | --- |
|  | HR (95% CI) | P-value | HR (95% CI) | P-value | HR (95% CI) | P-value | HR (95% CI) | P-value |
| ELSA |  |  |  |  |  |  |  |  |
| CES-D |  |  |  |  |  |  |  |  |
| 0 | 1.00 (Reference) | - | 1.00 (Reference) | - | 1.00 (Reference) | - | 1.00 (Reference) | - |
| 1-2 | 1.18 (0.94 ~ 1.48) | 0.143 | 1.19 (0.95 ~ 1.50) | 0.135 | 1.19 (0.95 ~ 1.50) | 0.131 | 1.20 (0.95 ~ 1.50) | 0.128 |
| 3-4 | 1.45 (1.08 ~ 1.94) | 0.013 | 1.31 (0.97 ~ 1.76) | 0.078 | 1.29 (0.96 ~ 1.74) | 0.093 | 1.30 (0.97 ~ 1.76) | 0.081 |
| >=5 | 1.59 (1.20 ~ 2.10) | 0.001 | 1.52 (1.14 ~ 2.02) | 0.004 | 1.51 (1.13 ~ 2.01) | 0.005 | 1.51 (1.13 ~ 2.01) | 0.005 |
| HRS |  |  |  |  |  |  |  |  |
| CES-D |  |  |  |  |  |  |  |  |
| 0 | 1.00 (Reference) | - | 1.00 (Reference) | - | 1.00 (Reference) | - | 1.00 (Reference) | - |
| 1-2 | 1.28 (1.13 ~ 1.45) | <.001*** | 1.27 (1.12 ~ 1.43) | <.001*** | 1.26 (1.12 ~ 1.43) | <.001*** | 1.25 (1.11 ~ 1.42) | <.001*** |
| 3-4 | 1.89 (1.62 ~ 2.20) | <.001*** | 1.70 (1.45 ~ 1.98) | <.001*** | 1.68 (1.44 ~ 1.97) | <.001*** | 1.61 (1.37 ~ 1.88) | <.001*** |
| >=5 | 2.03 (1.73 ~ 2.38) | <.001*** | 1.85 (1.57 ~ 2.19) | <.001*** | 1.83 (1.54 ~ 2.16) | <.001*** | 1.75 (1.48 ~ 2.07) | <.001*** |
| Model 1: Crude | | | | | | | | |
| Model 2: Adjust: Age, Sex, Race, Education, marital status, Wealth, BMI | | | | | | | | |
| Model 3: Adjust: Age, Sex, Race, Education, marital status, Wealth, BMI, Hypertension, Diabetes, Drinking status, Smoking status | | | | | | | | |
| Model 4: Adjust: Age, Sex, Race, Education, marital status, Wealth, BMI, Hypertension, Diabetes, Drinking status, Smoking status, Physical activity, Cardiovascular disease | | | | | | | | |

Supplementary Figure 1. Unadjusted mediation analysis of the association between depressive symptoms and incident ADL disability via physical activity: (A) ELSA cohort; (B) HRS cohort.


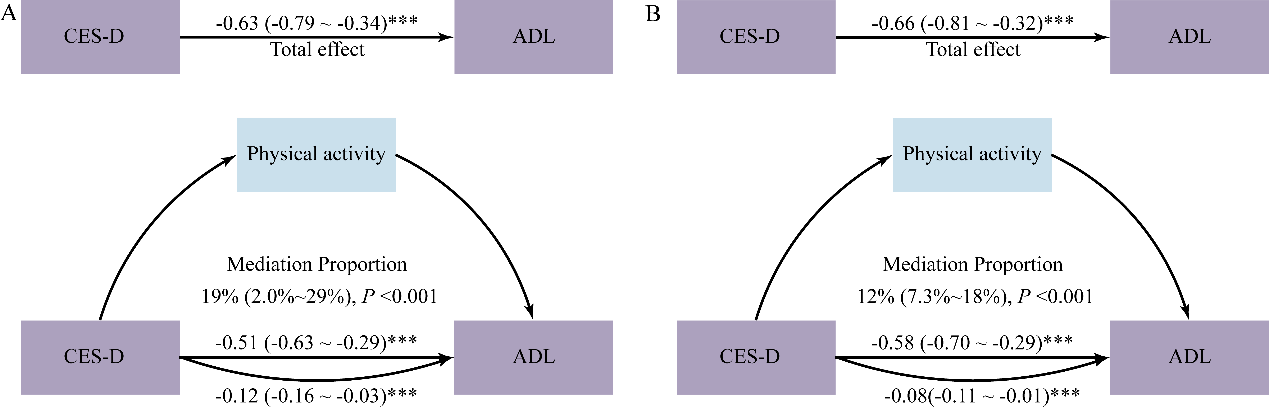

Supplement: Supplementary file 1 [file Data_Sheet_1.docx]
